# Evaluation of Passive Silicone Samplers Compared to Active Sampling Methods for Polycyclic Aromatic Hydrocarbons During Fire Training

**DOI:** 10.3390/toxics13020132

**Published:** 2025-02-12

**Authors:** Paro Sen, Miriam Calkins, Keith Stakes, Danielle L. Neumann, I-Chen Chen, Gavin P. Horn

**Affiliations:** 1 National Institute for Occupational Safety & Health, Cincinnati, OH 45213, USA; mcalkins@cdc.gov (M.C.); okv0@cdc.gov (I.-C.C.); 2 Fire Safety Research Institute, UL Research Institutes, Columbia, MD 21045, USA; keith.stakes@ul.org (K.S.); gavin.horn@ul.org (G.P.H.); 3 Enterprise Sustainability, UL Solutions, Brea, CA 92821, USA; summer.neumann@ul.com

**Keywords:** firefighter, PAH, wristband, passive sampling, silicone

## Abstract

Firefighters are occupationally exposed to many chemicals, including polycyclic aromatic hydrocarbons (PAHs), which are formed by the incomplete combustion of organic matter during fire response and training activities. However, due to the harsh environments in which firefighters work, as well as consideration for time and physical safety while wearing bulky equipment, traditional active sampling methods may not be feasible to measure PAH exposures. Silicone passive samplers offer an alternative approach to assess exposure during fire responses and live fire training due to their heat resistance and ease of deployment in remote or time-limited environments. In this study, the primary objective was to investigate and determine the statistical strength of the relationship between active air sampling methods and passive silicone samplers for PAHs. In this study, silicone wristbands were paired with active sampling devices in a series of burn experiments to compare PAH measurements. Silicone-based measurements correlated strongly with active air samples for the dominant PAHs found, naphthalene and phenanthrene; however, detection was limited in the wristbands when air concentrations were low in active samples. In situations where PAH levels are expected to be high and the potential for contaminant loss via off-gassing is low, silicone samplers may be a useful tool for industrial hygienists to measure PAHs in fire and other emergency responses in extreme environments.

## 1. Introduction

Occupational exposures experienced during firefighting are complex and multifaceted; however, exposure to polycyclic aromatic hydrocarbons (PAHs), a group of chemicals formed by the incomplete combustion of organic matter which includes naphthalene, phenanthrene, and pyrene, has been documented due to fire response, training, investigation, and other fire service tasks as well as in environments such as the fire apparatus and fire stations [1,2,3]. The International Agency for Research on Cancer (IARC) classified occupational exposure as a firefighter as carcinogenic to humans (Group 1) and noted the wide range of chemicals (including PAHs) firefighters are exposed to while working [4]. Because of the wide range of tasks and activities firefighters perform while working, it is challenging to delineate what a firefighter’s typical exposure profile looks like without more data. Thus, further exposure monitoring is necessary to better understand the sources and variability of PAH exposures that firefighters may face.

Several PAHs have also been categorized regarding their carcinogenic potential by IARC. Benzo[a]pyrene has been classified as a Group 1 carcinogen, while dibenzo[a,h]anthracene is a probable human carcinogen (Group 2A), and benzo[a]anthracene, benzo[b]fluoranthene, benzo[k]fluoranthene, chrysene, indeno [1,2,3-cd]pyrene, and naphthalene are possible human carcinogens (Group 2B). These classifications are based on evidence of carcinogenicity found in studies of animals and human cells [5]. Human studies of exposure to PAHs are more complex because PAHs are typically found in the environment in mixtures, so it is difficult to identify the impacts of individual chemicals on health. Major anthropogenic sources of PAHs include processes where coal or coal-based products, such as coal tar, are burned, as well as the combustion or incineration of waste and other material, vehicle exhaust, grilled foods, and cigarette smoke [5]. Studies have shown that PAHs eliminate relatively quickly from the body, typically within 1–2 days. A study of urinary PAH levels after eating barbecued chicken found that urinary PAH levels peaked three to five hours after eating and returned to baseline within 24–48 h [6]. A study on firefighters’ PAH levels in urine post-fire response showed similar results; PAH concentrations peaked three hours post-exposure for most tasks and remained elevated (although at much lower levels) 23 h later [7].

Occupational exposure limits (OELs) for PAHs are available for coal tar pitch solids, which includes anthracene, benzo(a)pyrene, chrysene, phenanthrene, and pyrene under one OEL. The Occupational Safety and Health Administration (OSHA) permissible exposure limit (PEL) and the American Conference of Governmental Industrial Hygienists (ACGIH) threshold limit value (TLV) are 0.2 milligrams per cubic meter of air (mg/m^3^), and the National Institute for Occupational Safety and Health (NIOSH) recommended exposure limit (REL) for coal tar pitch volatiles is 0.1 mg/m^3^ [8]. These limits represent air concentrations that a worker may be exposed to, typically during an 8–10 h shift, 40 h a week, for a working lifetime, without experiencing adverse health effects. To accompany the TLV for coal tar pitch solids, ACGIH has also established a biological exposure index (BEI) for PAHs of 2.5 micrograms per liter (µg/L) of the metabolite 1-hydroxypyrene in urine collected at the end of the working week. This level represents the concentration that would be found in healthy workers [9].

To measure contaminants in the air, “active” methods that utilize a sampling pump to draw in air to be analyzed for contaminants of interest are often considered the gold standard of personal air sampling, such as in the NIOSH Method 5528 for PAHs [10]. However, firefighters often work in extreme environments where conditions, such as high soot density, temperature, and/or humidity, may cause an electronic pump to malfunction, rendering active air sampling difficult [1]. For this reason, passive sampling devices that measure airborne chemical concentrations by capturing contaminants from the air on the sampling medium have grown in popularity as an alternative method of assessing vapor exposures experienced by the fire service. These devices are typically easy to deploy as they do not require a battery or calibration, are lightweight, and are able to withstand the wide range of environments in which firefighters work. Traditionally, passive sampling devices use charcoal media; however, other media have been used with increasing popularity in some settings. Silicone, especially in the form of a wearable wristband, is one such media that has gained interest for its ability to collect many semi-volatile organic compounds (SVOCs) for analysis. In the past few years, silicone passive samplers have been integrated into firefighters’ exposure assessments on several occasions [11,12,13,14,15,16,17,18].

Although many PAHs have been detected in silicone samplers, most commonly in the form of wristbands or dogtags worn on the wrist or around the neck, the results are typically reported as mass of contaminant per mass of sampling media and are not readily translatable to air concentrations. The relationship between contaminant mass adsorbed to a silicone sampler and the concentration in air is governed by partitioning of the contaminant between these two media. While the flow rate of air across the media is regulated in an active device, in passive samplers, it may be affected by environmental factors such as temperature, humidity, and turbulence of the air in the vicinity of the sampler [16]. However, if strong correlations are found between concentrations measured by active air sampling methods and those in the passive silicone samplers, it may be possible to apply those correlations to estimate air concentrations from silicone sampler measurements in similar environments.

In this study, the primary objective was to investigate and determine the statistical strength of the relationship between active air sampling methods and passive silicone samplers for a group of chemicals with high relevance to firefighter exposures. To accomplish this objective, the research team measured PAH concentrations using active samples from the sorbent phase of an OSHA Versatile Sampler (OVS) tube and passive silicone wristbands during a series of controlled training fire experiments. The research team hypothesized that a statistically significant, positive relationship exists between measurements of PAHs collected on active air sampling media and silicone wristbands used as passive samplers when paired together in a fire training environment.

## 2. Materials and Methods

### 2.1. Sampling Procedures

Two types of sampling devices were used to measure PAH exposures: passive silicone wristbands (24HourWristbands.com; Houston, TX, USA) and active air sampling via NIOSH method 5528 utilizing an XAD-7 sorbent/glass fiber filter OVS tubes [19]. The passive silicone samplers, often referred to by their “wristband” shape, were black in color and approximately 6 millimeters (mm) wide and 2 mm thick. The average weight of the wristbands was 4.55 g. Prior to deployment, wristbands were prepared for use following the methods used in O’Connell et al. using three rounds of extraction with a 1:1 mixture of ethyl acetate and hexane, two rounds with a 1:1 mixture of ethyl acetate and methanol, and drying overnight in a vacuum oven to remove excess solvent. Wristbands were stored in sealed amber glass vials following cleaning, including during transport to the live fire training site and to the laboratory [20].

The sampling devices were deployed during 15 controlled live fire training experiments at a fire training facility in Gunpowder, Maryland. A detailed description of the fire experiments can be found in Horn et al. [19]. The live fire-training experiments occurred in a concrete multistory training structure with two floors and five rooms. Each fire commenced in the same first floor room. Three different wood-based fuel loads (oriented strand board (OSB), fiberboard, and/or solid wood pallets) along with straw were utilized, with five replicates of each fuel load. Differences between fuel types are reported for the full experiment by Horn et al. [19]; due to the small sample size for the experiments with wristbands, we were unable to draw robust comparisons between each fuel type. The two sampling devices were located in the second floor hallway representing where an instructor would be located [19]. The OVS tubes connected to air sampling pumps (Gilian BDX-II [Sensidyne, St. Petersburg, FL, USA] or PCXR4 Universal sample pumps [SKC, Eighty Four, PA, USA]) and the passive silicone wristbands were situated on a stationary apparatus roughly 0.9 m above the floor, which was an approximation of the head height of a kneeling or crouching instructor. The sampling pump was located inside an insulated cabinet and the inlet of the OVS tube extended outside the cabinet. The pump was pre- and post-calibrated to a flow rate of 1 L of air per minute (L/min). The wristbands were hung outside of the cabinet that contained the sampling pumps adjacent to the OVS tubes.

Two minutes prior to ignition, the sampling pumps were turned on and the wristbands were removed from the amber bottle using clean gloves and tweezers. The jar was subsequently closed to avoid picking up additional contamination. Each fire lasted for approximately 16 min. Two minutes after the fire suppression was complete, firefighters would enter the training structure to remove the cabinet that contained the sampling devices. Sample pumps were turned off, and OVS tubes were capped and wrapped in aluminum foil. Wristbands were collected using clean gloves and tweezers and returned to the amber glass jars. The timing of when the media was exposed was recorded. The OVS tubes were stored in a refrigerator at approximately 4 °C pre- and post-sampling and on ice packs during shipping to the analytical laboratory under chain of custody. One OVS tube and one wristband were submitted to the laboratory for each of the 15 fire experiments. One OVS field blank was collected on each of the six days of sampling [19]. Two silicone wristband field blanks were also collected, one on the first and one on the last day of sampling.

After sampling, the wristbands were stored in amber glass jars at room temperature and shipped overnight to the analytical laboratory maintaining chain of custody. Wristbands were stored by the laboratory for up to three weeks prior to desorption and analysis following methods used by O’Connell et al. [20]. Wristbands were prepared for analysis by rinsing with deionized water and isopropyl alcohol to remove surface debris. They were then desorbed into ethyl acetate and analyzed by gas chromatography/mass spectrometry (GC/MS) for the 16 PAHs designated as priority pollutants by the U.S. Environmental Protection Agency [21] as well as 1- and 2-methylnaphthalene, for a total of 18 PAHs (Table 1). The OVS tubes were prepared and analyzed following NIOSH Method 5528 for the 16 priority pollutant PAHs listed in Table 1. While 1- and 2-methylnaphthalene were analyzed in wristbands, they are not included in NMAM Method 5528 and thus were not included in the analysis of OVS tubes. The limit of detection (LOD) for each individual PAH was 0.5 micrograms (μg) per wristband, or 0.11 μg/g of silicone, and 0.08–0.2 μg per OVS sample. OVS field blanks during three of the fifteen experiments had naphthalene levels above the LOD on the sorbent portion, and the corresponding samples were background corrected to account for this. Background levels were two orders of magnitude lower than the levels measured during the experiments. OVS data were analyzed to determine the amount of contaminant measured on the filters (representing the particle phase of PAHs) and the sorbents (representing the vapor phase). Wristbands analyzed using this method are only expected to measure the vapor phase, so only the sorbent data from the OVS tubes were used for comparison between media.

### 2.2. Data Analysis

Summary statistics including detection frequency, median, and range, were calculated for all PAHs. Further statistical analysis was only conducted on those chemicals where detection frequencies were greater than 60% for both media. Data were log-transformed to account for the right-skewed nature of exposure data created by the inherent variability of fires, and geometric means and standard deviations were calculated as well. Values below the LOD were imputed using the beta-substitution method [22]. For chemicals with higher detection frequencies, correlation tests were used to evaluate the performance of wristbands compared to the OVS sorbents because the units of the two measurements are different. Wristband data are reported in mass of analyte per gram of silicone (μg/g) as opposed to mass per air volume (micrograms per cubic meter of air (μg/m^3^)) for samples collected on OVS sorbents. Masses collected on wristbands were normalized by the average mass of the wristbands (4.55 g) to facilitate better comparison to other wristband studies. Statistical analyses were conducted in R version 4.3.2 [23].

## 3. Results

Total PAH concentrations in active samples (both filter and sorbent) ranged from 270 μg/m^3^ to 10,400 μg/m^3^. Total PAH concentrations ranged from below the limit of detection to 1530 μg/m^3^ and 260 to 9200 μg/m^3^ on the filter and sorbent media, respectively. Comparisons to wristbands are limited to just the sorbent sections. Table 1 provides the detection frequencies and descriptive statistics of the XAD-7 sorbent section of the OVS tubes and wristbands. Thirteen out of the sixteen PAHs analyzed in active air samples were measured in the sorbent section of at least one OVS tube, and ten out of eighteen PAHs analyzed in silicone were measured in at least one wristband. All three of the PAHs not measured in the sorbent (benzo[g,h,i]perylene, benzo[k]fluoranthene, and dibenzo[a,h]anthracene) were detected in the filter of the OVS tube and were not found in the wristbands. Over 90% of the contaminant mass measured by the OVS tube was found on the sorbent section, while less than 10% was found in the filter. More information about the distribution between the particle and vapor phase measurements is available in Supplementary Table S2.

Across the 15 fire experiments, naphthalene and phenanthrene were detected in all wristband and sorbent samples and were consistently measured at higher concentrations than other PAHs. Fluoranthene and acenaphthylene were also detected in all sorbents and most wristbands (60% and 73%, respectively). However, the passive silicone wristband samplers did not appear to be as sensitive to lower concentrations as the OVS sorbent, as they typically had low detection frequencies for compounds with low average concentrations in active samples, and detection limits for wristbands were higher than in the XAD-7 sorbent per sample (0.5 μg/band vs. 0.08–0.2 μg/sample). Anthracene, pyrene, and fluorene were each detected in 93% of the sorbent tubes with relatively low concentrations but were detected in fewer than half of the wristband samples. Additionally, acenaphthene was detected in just under half of the sorbents and only one wristband sample. The other eight PAHs evaluated in both wristbands and OVS tubes were not detected in any of the wristbands and only one or two of the sorbent samples (benzo[a]pyrene, benz[a]anthracene, benzo[b]fluoranthene, benzo[k]fluoranthene, benzo[g,h,i]perylene, chrysene, dibenz[a,h]anthracene, and indeno [1,2,3-c,d]pyrene). The two PAHs only analyzed in the wristbands, 1- and 2-methylnapthalene, were each detected in 60% of wristband samples.

**Figure 1:**
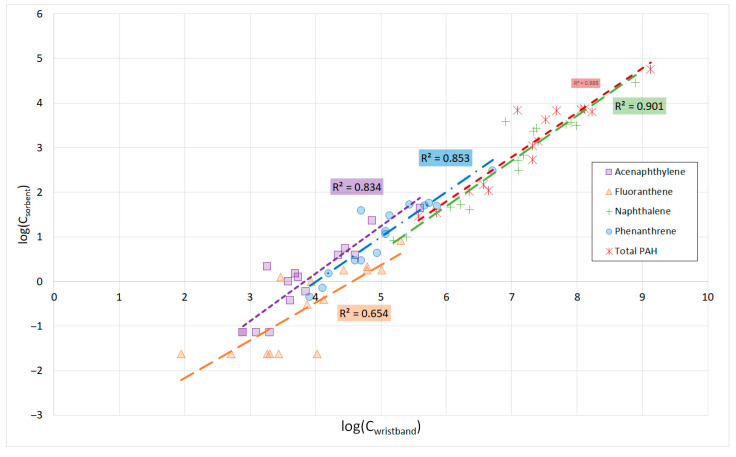
Pearson correlations between log-transformed OVS sorbent and wristband data for acenaphthylene, fluoranthene, naphthalene, phenanthrene, and total PAH.

We evaluated correlations between log-transformed data for the compounds detected in both sampling media types at frequencies of 60% or higher, as follows: acenaphthylene, fluoranthene, naphthalene, and phenanthrene. Figure 1 presents the Pearson correlation test results between the log-transformed OVS sorbent measurements and the wristband data. All four chemicals for which correlations were analyzed had significant effects (*p* < 0.001) with correlation coefficients greater than 0.8, indicating strong relationships between the OVS sorbent measurements and those from the wristbands. Pearson and Spearman correlation coefficients can be found in Supplementary Table S3.

## 4. Discussion

To our knowledge, this is the first study in which the strength of the relationship is assessed between active and passive sampling measurements of PAHs in live fire environments. Dixon et al. reported correlations between backpacks containing active air sampling devices and silicone wristbands worn by a cohort of pregnant women in New York City, but the correlations and significances found were lower overall than in this study [24]. Dixon et al. reported significant (*p* < 0.05) Spearman correlations of 0.56, 0.71, and 0.54 for fluoranthene, naphthalene, and phenanthrene compared to Spearman correlations of 0.87, 0.85, and 0.93 for the same three compounds in this study, respectively. The stronger correlations in our study may be a result of the higher air concentrations of PAHs in live fires, yielding higher detection frequencies.

In this study, the range of exposures measured by active sampling devices is well within those reported by other exposure assessments of fire instructors in live fire-training scenarios. In this study, total PAH concentrations on both sorbent and filter ranged from 270 to 10,400 μg/m^3^ for the area air measurements collected on the OVS tube, with a median concentration of 1590 μg/m^3^. A 2015 study of fire instructors during structural firefighting training activities found total PAH concentrations ranging from 430 μg/m^3^ to 2700 μg/m^3^ in personal samples collected on the outside of instructors’ turnout gear [25]. Fent et al. collected personal air samples from instructors supervising teams during training scenarios with results ranging from 1230 to 19,900 μg/m^3^ [26]. Keir et al. reported that firefighters suppressing training fires in shipping containers had total PAH exposures ranging from 200 to 3970 μg/m^3^ [27]. Fent et al. also collected personal air samples from firefighters performing suppression activities during training exercises with total PAH measurements ranging from 130 μg/m^3^ to 22,000 μg/m^3^ [1]. These exercises were both performed in relatively small single-story structures, with one made of drywall, with a median total PAH concentration of 5300 μg/m^3^, and a metal structure, with a median total PAH of 1400 μg/m^3^. Kirk and Logan and Keir et al. included the same 16 PAHs as we did in their analyses and measurements of total PAHs [25,27]; Fent et al. also included benzo(e)pyrene in analyses for a total of 17 PAHs in 1 study [1], but they excluded acenaphthylene in another for a total of 15 PAHs [26].

The differences in available analytical methods, the wide variety of fuels and training structures, the inherent unpredictability of fires, and differences in sampling location and methodology all contribute to the differences in exposure profiles between this study and others in the past. The available literature represents a wide range of training scenarios, structure designs, ventilation conditions, and fuel packages. Furthermore, analytical methods have evolved considerably in recent years. NIOSH Method 5528, used in this study, was published in 2021 and offers improved recoveries of PAHs, particularly naphthalene, the most volatile PAH, from OVS tubes than prior methods. For naphthalene, the older NIOSH Method 5506 reported a recovery of 49.6% from filters and 68.5% from sorbents [28], compared to 85.7–110% in the more recent NIOSH Method 5528 [10]. NIOSH Method 5506 employs high performance liquid chromatography analysis of OVS tubes containing PTFE filters and XAD-2 sorbent, while NIOSH Method 5528 uses gas chromatography to analyze OVS tubes with glass fiber filers and XAD-7 sorbent. These differences among others likely impact the improvement in performance of NIOSH Method 5528 from the prior published methods.

Wristbands and other silicone-based sampling devices have been used to quantify firefighters’ exposure to PAHs several times before. Two previous studies have used wristbands to measure PAHs in fire training scenarios. Keir et al. measured the differences in PAH concentrations on the neck outside of turnout gear and on the wrist under the turnout gear using silicone wristbands [16]. Sampling times ranged from 39 and 63 min, and total PAHs measured from the bands ranged from 0.54 to 5.8 micrograms per gram (μg/g) of silicone, with a geometric mean of 1.8 μg/g. We measured a much wider range of exposure spanning from 0.87 μg/g to 24.2 μg/g with a geometric mean of 4.4 μg/g. The differences in ranges are consistent with our air sampling results (both sorbent and filter) spanning a wider range of concentrations (270 to 10,400 μg/m^3^) than the active sampling data reported by Keir et al. for the same experiments (200 to 3970 μg/m^3^) [27]. Bonner et al. also used silicone wristbands to evaluate the protection from fireground smoke provided by turnout gear with a sampling time of only 10 min [14]. They reported average airborne naphthalene measurements of 60.6 μg/g in the abdominal sampling location outside of the turnout gear (the closest to the sampling height of 0.9 m in our study), compared to an average concentration of 5.1 μg/g in this study. The higher concentrations found by Bonner et al. are likely due to different fuel packages (i.e., upholstered sofa vs. wood products), and wristband location (i.e., sampling in a smaller training structure closer to the fire may offer more exposure than sampling in a larger training structure on a different floor from the fire). The airflow at the location of the silicone samples in our study with a large multi-story structure was likely dramatically different than in Bonner et al. where samplers were used in a relatively small, single-story structure, which could affect the uptake of contaminants by the sampling devices. In each of these studies, naphthalene was the dominant compound measured.

However, two other studies have characterized firefighters’ exposures using silicone sampling media and substantially longer sampling times. Poutasse et al. instructed firefighters to wear silicone dogtags continuously for a total of 60 days (30 on-shift and 30 off-shift) and found 2-methylnaphthalene and phenanthrene to be the dominant PAHs [18]. The researchers reported a mean concentration of less than 0.05 μg/g silicone for 2-methylnaphthalene and less than 0.03 μg/g for naphthalene. Similarly, Levasseur et al. instructed participants to wear wristbands continuously during a six-day shift and reported a detection frequency of less than 15% for naphthalene [17]. The differences in concentrations and detection frequencies between these studies and ours may be caused by several reasons. Participants in some previous studies may have worn sampling devices beneath PPE during fire responses, resulting in lower measurements. Long sampling times including periods of time outside of active response may also yield lower concentrations measured on silicone samplers. In situations where firefighters wear silicone sampling devices during intermittent periods of exposure to higher and lower PAH concentrations (e.g., higher exposures during fire response and lower exposures working in the fire station), there may be the opportunity for PAHs adsorbed to the silicone, particularly naphthalene (the most volatile PAH), to diffuse back into a lower exposure environment. The off-gassing of PAHs from exposed firefighting gear to private vehicles and changing rooms of fire stations has been demonstrated in prior studies [3,29]. Given that silicone passive samplers operate via diffusion of chemicals from areas of high to low concentrations, loss of contaminants may be possible when these samplers are worn in environments with low concentrations following training or response activities. A study evaluating uptake of contaminants by silicone wristbands worn by a convenience sample of 10 adults over a period of 5 days also found that the concentration of naphthalene in the samplers decreased as sampling time increased. The opposite was true for other PAHs; the concentration and sampling time were positively correlated [30]. Based on the results of our study as well as those reported by Bonner et al. and Keir et al. [14,16], for measuring PAHs, especially naphthalene, silicone passive samplers appear to perform better when used in environments with potential for consistent high exposures.

For naphthalene and phenanthrene, the compounds measured at the highest concentrations by the OVS sorbent, the wristbands were able to quantify levels of these compounds across all 15 fire experiments and yield strong correlations between the two media. However, lack of sensitivity to lower concentrations of PAHs is a limitation of the current analytical methods for silicone passive samplers. For compounds measured at low concentrations where active sampler medians fell below approximately 30 μg/m^3^, the wristbands detected them at lower frequencies. These low detection frequencies prevented the research team from drawing robust statistical conclusions about the performance of wristbands for these compounds. Even for two of the compounds for which the research team did analyze correlations, the wristbands were not able to detect contaminants at the same frequencies as the OVS sorbents—for example, fluoranthene was detected in every OVS sorbent, but only nine of the fifteen wristbands, and with a weaker correlation coefficient.

Firefighters are exposed to a complex mixture of contaminants including PAHs and many other groups of chemicals [4]. While the results of this study indicate measurements from active air monitoring and passive silicone wristbands are strongly associated for certain PAHs when present at higher concentrations, evaluation of the association for other chemical groups and their respective methods was beyond the scope of this study. Silicone sampling devices have been used to detect numerous chemical groups in studies involving firefighters, including phthalates, flame retardants, and pesticides [18]. Evaluating the relationship between active air sampling methods and passive sampling using silicone for these compounds is an opportunity for future research to further the potential of silicone media as a tool to assess firefighters’ exposures.

Silicone wristbands and other sampling devices appear to have promise in situations where exposures to PAHs are known to be high and where the potential for off-gassing is limited. Compared to traditional active sampling methods, the media is typically much lower in cost and easier to deploy in time and resource constrained environments, such as emergency responses, since they do not require equipment that must be charged, calibrated, and tested immediately prior to use. In some research settings, participants have mailed sampling devices back to researchers after wearing them, allowing researchers to collect data remotely, because silicone sampling devices such as wristbands are lightweight and easy to store and ship [30]. Passive sampling devices are also less cumbersome than active sampling devices, which require the use of an air pump, so researchers can use them to collect measurements from firefighters who already wear bulky personal protective equipment. The low cost and ease of use also make it possible to use these sampling devices to collect measurements without a trained industrial hygienist’s supervision in the field. These factors have enabled researchers to use silicone media to collect data from more diverse populations and larger sample sizes than the constraints of active air sampling techniques would allow [31]. However, more research is needed to consistently derive air concentrations from PAH measurements in silicone samplers—currently, results are typically reported as mass of contaminant per mass of silicone, whereas more information about contaminant behavior and the impact of temperature and humidity is necessary to convert these measurements to mass of contaminant per air volume.

## 5. Conclusions

Overall, silicone passive sampling devices such as wristbands show promise in assessing firefighters’ exposures to PAHs when concentrations are high and losses from off-gassing are unlikely. The results of this experiment demonstrate that silicone sampler measurements for PAHs found in abundance in the vapor phase correlated well with sorbent measurements collected using active air sampling methods. However, in this study, we observed that wristbands were not as sensitive to low PAH concentrations compared to active samplers. Further research is needed to evaluate the performance and improve the sensitivity of silicone passive sampling devices for compounds found at lower concentrations than naphthalene (GM: 1070 μg/m^3^) and phenanthrene (GM: 157 μg/m^3^).

Although the correlations reported between active and passive sampling devices in this study were quite strong for the four compounds evaluated, more research is required to translate silicone sampler measurements into air concentrations that could then be compared with existing occupational exposure limits. A greater understanding of factors such as exposure time, humidity, temperature, and air movement on silicone sampler performance is needed to further develop this as a sampling method for PAHs to be comparable to active air methods. However, silicone samplers do offer advantages such as ease of deployment, low cost, and lack of electronic components that make them ideal for use in the harsh environments in which firefighters often work. For tasks with high exposure levels, silicone passive samplers may have potential as a screening method or as a means of assessing exposures to PAHs faced by firefighters and reducing the risk of adverse health effects in the profession.

## Figures and Tables

**Table 1:** Descriptive statistics of vapor phase measurements in XAD-7 sorbent section of OSHA Versatile Samplers and silicone wristband samples of 18 polycyclic aromatic hydrocarbons (PAHs) during 15 live fire-training experiments.

Compound ^a^	XAD-7 Sorbent			Wristband		
	Detection Frequency	Median Air Concentration ^c^ (Minimum; Maximum)	Geometric Mean ^f,g^ (Geometric Standard Deviation)	Detection Frequency	Median Sample Mass (Minimum; Maximum)	Geometric Mean (Geometric Standard Deviation)
	% (*n*)	μg/m^3^	μg/m^3^	% (*n*)	μg/g Wristband	μg/g Wristband
Acenaphthene	47 (7)	N/A (<LOD ^d^; 33)	--	7 (1)	N/A (<LOD ^e^; 0.13)	-
Acenaphthylene	100 (15)	40 (18; 270)	47 (2.18)	73 (11)	0.24 (<LOD; 1.1)	0.22 (2.47)
Anthracene	93 (14)	11 (<LOD; 70)	11.7 (2.2)	13 (2)	N/A (<LOD; 0.19)	-
Benzo(a)anthracene	7 (1)	N/A (<LOD; 7.7)	-	0 (0)	-	-
Benzo(a)pyrene	7 (1)	N/A (<LOD; 6.5)	-	0 (0)	-	-
Benzo(b)fluoranthene	13 (2)	N/A (<LOD; 12)	-	0 (0)	-	-
Benzo(g,h,i)perylene	0 (0)	-	-	0 (0)	-	-
Benzo(k)fluoranthene	0 (0)	-	-	0 (0)	-	-
Chrysene	7 (1)	N/A (N/A; 9.5)	-	0 (0)	-	-
Dibenzo(a,h)anthracene	0 (0)	-	-	0 (0)	-	-
Fluoranthene	100 (15)	51 (7; 200)	49 (2.47)	60 (9)	0.15 (<LOD; 0.55)	0.12 (2.59)
Fluorene	93 (14)	11 (<LOD; 74)	12 (2.6)	27 (4)	N/A (<LOD; 0.29)	
Indeno(1,2,3-cd)pyrene	7 (1)	N/A (<LOD; 7.7)	-	0 (0)	-	-
Naphthalene	100 (15)	1200 (180; 7300)	1100 (2.73)	100 (15)	3.7 (0.55; 19)	3.2 (2.94)
Phenanthrene	100 (15)	160 (50; 820)	160 (2.11)	100 (15)	0.68 (0.16; 2.6)	0.63 (2.26)
Pyrene	93 (14)	30 (<LOD; 120)	29 (2.69)	47 (7)	N/A (<LOD; 0.37)	--
Total PAH ^b^	-	1500 (260; 9200)	1400 (2.58)	-	4.9 (0.87; 24)	4.4 (2.75)

^a^ 1- and 2-methylnaphthalene were not analyzed in OVS tubes, only wristbands; data appear in Table S1. ^b^ Total PAH includes imputed values for compounds where concentration was below the limit of detection (LOD). ^c^ Median was below the LOD for any compound with detection frequency of 7 or lower, represented by “N/A”. ^d^ LOD for OVS tubes ranged from 0.08 to 0.2 μg/sample depending on compound. Volume collected (m^3^) per sample depends on pump run time, which varied slightly between experiments. ^e^ LOD for wristbands was 0.11 μg/g silicone. ^f^ Geometric means were only calculated for those compounds with detection frequencies >60%. ^g^ Geometric means and standard deviations include imputed values where detection frequencies <100%.

## Data Availability

The data that support the findings of this study are available from the corresponding author upon reasonable request.

## Supplementary Materials

The following supporting information can be downloaded at: https://www.mdpi.com/article/10.3390/toxics13020132/s1, Table S1: Concentrations of 1- and 2-Methylnaphthalene on silicone wristband samples; Table S2: Distributions of Compounds Between Filter and Sorbent on Active Samples; Table S3: Correlation Coefficients (r) Between Sorbent and Wristband for Selected Compounds.

## References

[1] Fent K.W., Eisenberg J., Snawder J., Sammons D., Pleil J.D., Stiegel M.A., Mueller C., Horn G.P., Dalton J. (2014). Systemic Exposure to PAHs and Benzene in Firefighters Suppressing Controlled Structure Fires. Ann. Occup. Hyg..

[2] Horn G.P., Fent K.W., Kerber S., Smith D.L. (2022). Hierarchy of contamination control in the fire service: Review of exposure control options to reduce cancer risk. J. Occup. Environ. Hyg..

[3] Rogula-Kozłowska W., Bralewska K., Rogula-Kopiec P., Makowski R., Majder-Łopatka M., Łukawski A., Brandyk A., Majewski G. (2020). Respirable particles and polycyclic aromatic hydrocarbons at two Polish fire stations. Build. Environ..

[4] Demers P.A., DeMarini D.M., Fent K.W., Glass D.C., Hansen J., Adetona O., Andersen M.H., Freeman L.E.B., Caban-Martinez A.J., Daniels R.D. (2022). Carcinogenicity of occupational exposure as a firefighter. Lancet Oncol..

[5] Jameson C.W., Baan R.A., Stewart B.W., Straif K. (2019). Polycyclic aromatic hydrocarbons and associated occupational exposures. Tumour Site Concordance and Mechanisms of Carcinogenesis. International Agency for Research on Cancer.

[6] Li Z., Romanoff L., Bartell S., Pittman E.N., Trinidad D.A., McClean M., Webster T.F., Sjödin A. (2012). Excretion Profiles and Half-Lives of Ten Urinary Polycyclic Aromatic Hydrocarbon Metabolites after Dietary Exposure. Chem. Res. Toxicol..

[7] Fent K.W., Toennis C., Sammons D., Robertson S., Bertke S., Calafat A.M., Pleil J.D., Wallace M.A.G., Kerber S., Smith D. (2020). Firefighters’ absorption of PAHs and VOCs during controlled residential fires by job assignment and fire attack tactic. J. Expo. Sci. Environ. Epidemiol..

[8] NIOSH (2019). Coal Tar Pitch Volatiles.

[9] (2017). Polycyclic Aromatic Hydrocarbons (PAHs).

[10] NIOSH (2021). Polynuclear Aromatic Hydrocarbons in Air by GC-MS SIM: Method 5528.

[11] Bakali U., Baum J.L.R., Killawala C., Kobetz E.N., Solle N.S., Deo S.K., Caban-Martinez A.J., Bachas L.G., Daunert S. (2021). Mapping carcinogen exposure across urban fire incident response arenas using passive silicone-based samplers. Ecotoxicol. Environ. Saf..

[12] Bakali U., Baum J.L.R., Louzado-Feliciano P., Killawala C., Santiago K.M., Pauley J.L., Dikici E., Solle N.S., Kobetz E.N., Bachas L.G. (2024). Characterization of fire investigators’ polyaromatic hydrocarbon exposures using silicone wristbands. Ecotoxicol. Environ. Saf..

[13] Baum J.L.R., Bakali U., Killawala C., Santiago K.M., Dikici E., Kobetz E.N., Solle N.S., Deo S., Bachas L., Daunert S. (2020). Evaluation of silicone-based wristbands as passive sampling systems using PAHs as an exposure proxy for carcinogen monitoring in firefighters: Evidence from the firefighter cancer initiative. Ecotoxicol. Environ. Saf..

[14] Bonner E.M., Horn G.P., Smith D.L., Kerber S., Fent K.W., Tidwell L.G., Solle N.S., Deo S., Bachas L., Daunert S. (2023). Silicone passive sampling used to identify novel dermal chemical exposures of firefighters and assess PPE innovations. Int. J. Hyg. Environ. Health.

[15] Caban-Martinez A.J., Feliciano P.L., Baum J., Bakali U.F., Santiago K.M., Solle N.S., Rivera G., Ramirez C.E., Deo S., Miric M. (2020). Objective Measurement of Carcinogens Among Dominican Republic Firefighters Using Silicone-Based Wristbands. JCO Glob. Oncol..

[16] Keir J.L., Papas W., Wawrzynczak A., Aranda-Rodriguez R., Blais J.M., White P.A. (2023). Use of silicone wristbands to measure firefighters’ exposures to polycyclic aromatic hydrocarbons (PAHs) during live fire training. Environ. Res..

[17] Levasseur J.L., Hoffman K., Herkert N.J., Cooper E., Hay D., Stapleton H.M. (2022). Characterizing firefighter’s exposure to over 130 SVOCs using silicone wristbands: A pilot study comparing on-duty and off-duty exposures. Sci. Total Environ..

[18] Poutasse C.M., Poston W.S.C., Jahnke S.A., Haddock C.K., Tidwell L.G., Hoffman P.D., Anderson K.A. (2020). Discovery of firefighter chemical exposures using military-style silicone dog tags. Environ. Int..

[19] Horn G.P., Stakes K., Neumann D.L., Willi J.M., Chaffer R., Weinschenk C., Fent K.W. (2024). Chemical and Thermal Exposure Risks in a Multi Compartment Training Structure. Fire Technol..

[20] O’Connell S.G., Kincl L.D., Anderson K.A. (2014). Silicone Wristbands as Personal Passive Samplers. Environ. Sci. Technol..

[21] USEPA (2014). Priority Pollutant List.

[22] Ganser G.H., Hewett P. (2010). An accurate substitution method for analyzing censored data. J. Occup. Environ. Hyg..

[23] R Core Team (2023). R: A Language and Environment for Statistical Computing (Version 4.3.2).

[24] Dixon H.M., Scott R.P., Holmes D., Calero L., Kincl L.D., Waters K.M., Camann D.E., Calafat A.M., Herbstman J.B., Anderson K.A. (2018). Silicone wristbands compared with traditional polycyclic aromatic hydrocarbon exposure assessment methods. Anal. Bioanal. Chem..

[25] Kirk K.M., Logan M.B. (2015). Firefighting Instructors’ Exposures to Polycyclic Aromatic Hydrocarbons During Live Fire Training Scenarios. J. Occup. Environ. Hyg..

[26] Fent K.W., Mayer A., Bertke S., Kerber S., Smith D., Horn G.P. (2019). Understanding airborne contaminants produced by different fuel packages during training fires. J. Occup. Environ. Hyg..

[27] Keir J.L.A., Kirkham T.L., Aranda-Rodriguez R., White P.A., Blais J.M. (2023). Effectiveness of dermal cleaning interventions for reducing firefighters’ exposures to PAHs and genotoxins. J. Occup. Environ. Hyg..

[28] NIOSH (1998). Polynuclear Aromatic Hydrocarbons in Air by HPLC: Method 5506.

[29] Banks A.P.W., Wang X., He C., Gallen M., Thomas K.V., Mueller J.F. (2021). Off-Gassing of Semi-Volatile Organic Compounds from Fire-Fighters’ Uniforms in Private Vehicles-A Pilot Study. Int. J. Environ. Res. Public Health.

[30] Samon S.M., Hoffman K., Herkert N., Stapleton H.M. (2024). Chemical uptake into silicone wristbands over a five day period. Environ. Pollut..

[31] Dixon H.M., Armstrong G., Barton M., Bergmann A.J., Bondy M., Halbleib M.L., Hamilton W., Haynes E., Herbstman J., Hoffman P. (2019). Discovery of common chemical exposures across three continents using silicone wristbands. R. Soc. Open Sci..

