# Supplementary material for: Evaluation of Passive Silicone Samplers Compared to Active Sampling Methods for Polycyclic Aromatic Hydrocarbons During Fire Training
Source: Toxics. 2025 Feb 12;13(2):132. doi: 10.3390/toxics13020132 (PMC11860701; doi:10.3390/toxics13020132)
Supplement: Supplementary file 1 [file toxics-13-00132-s001.zip › toxics-3412825-supplementary.pdf]

Table S1: Concentrations of 1- and 2-Methylnaphthalene on silicone wristband samples

Compound	Detection Frequency	Median Sample Mass (Minimum; Maximum)	Geometric Mean (Geometric Standard Deviation)
	**% (n)**	**µg/g wristband**	**µg/g wristband**
1-Methylnaphthalene	60 (9)	0.12 (<LOD; 0.66)	0.11 (2.54)
2-Methylnaphthalene	60 (9)	0.13 (<LOD; 0.79)	0.12 (2.63)

Table S2: Distributions of Compounds Between Filter and Sorbent on Active Samples

Compound	Percent on Filter	Percent on Sorbent	Percent of Total PAH
Acenaphthene	0%	100%	0.3%
Acenaphthylene	0%	100%	2.9%
Anthracene	0%	100%	0.7%
Benzo(a)anthracene	96.9%	3.1%	0.7%
Benzo(a)pyrene	95.9%	4.1%	0.5%
Benzo(b)fluoranthene	94.4%	5.6%	1.0%
Benzo(g,h,i)perylene	100%	0%	1.9%
Benzo(k)fluoranthene	100%	0%	0.2%
Chrysene	96.3%	3.7%	0.8%
Dibenzo(a,h)anthracene	100%	0%	0.02%
Fluorene	0%	100%	0.8%
Fluoranthene	37.4%	62.6%	4.8%
Indeno(1,2,3-cd)pyrene	95.8%	4.2%	0.5%
Naphthalene	0%	100%	72.6%
Phenanthrene	0.4%	99.6%	9.1%
Pyrene	45.5%	54.5%	3.4%
Total PAH	8.8%	91.2%	100%

Table S3: Correlation Coefficients (r) Between Sorbent and Wristband for Selected Compounds

	Pearson r_p	Spearman r_s
Acenaphthylene	0.913	0.872
Fluoranthene	0.809	0.871
Naphthalene	0.949	0.846
Phenanthrene	0.924	0.929
Total PAH	0.946	0.893

All p-values were < 0.005. Correlation coefficients were calculated using log-transformed data due to the right-skewed nature of the sampling data.
